# Paclitaxel and concomitant radiotherapy in high-risk endometrial cancer patients: preliminary findings

**DOI:** 10.1186/1471-2407-6-198

**Published:** 2006-07-25

**Authors:** Giorgia Mangili, Patrizia De Marzi, Saverio Beatrice, Emanuela Rabaiotti, Riccardo Viganò, Luigi Frigerio, Cinzia Gentile, Ferruccio Fazio

**Affiliations:** 1 Gynecology Department, San Raffaele Hospital, Milan, Italy; 2 Radiotherapy Department, San Raffaele Hospital, Milan, Italy; 3 Gynecology Department Ospedali Riuniti, Bergamo, Italy; 4 CNR IBSM, University of Milano-Bicocca, Nuclear Medicine Department, San Raffaele Hospital, Milan, Italy

## Abstract

**Background:**

There is still much debate about the best adjuvant therapy after surgery for endometrial cancer (EC) and there are no current guidelines. Radiotherapy (RT) alone does not seem to improve overall survival. We investigated whether concomitant Paclitaxel (P) and RT gave better clinical results.

**Methods:**

Twenty-three patients with high-risk EC (stage IIB, IIIA, IIIC or IC G3 without lymphadenectomy or with aneuploid tumor) underwent primary surgery and were then referred for adjuvant therapy. P was given at a dose of 60 mg/m2 once weekly for five weeks during RT, which consisted of a total radiation dose of 50.4 Gy. Three further weekly cycles of P at a dose of 80 mg/m2 were given at the end of RT. Overall survival and disease-free survival were calculated from the time of surgery. Patterns of failure were recorded by the sites of failure.

**Results:**

A total of 157 cycles of P were administered both during radiotherapy and consolidation chemotherapy.

Relapses occurred in five patients (21.7%). Median time to recurrence was 18.6 months (range 3–28). Survival rate for all the patients was 78.2%. Overall survival for the patients who completed chemo-radiation was of 81%. In this group median time to recurrence was 19.2 months (range 3–28). All recurrences were outside the radiation field. Mortality rate was 14.2%.

**Conclusion:**

This small series demonstrates pelvic radiotherapy in combination with weakly P followed by three consolidation chemotherapy cycles as an effective combined approach in high risk endometrial carcinoma patients.

## Background

Patients with endometrial cancer (EC) are traditionally divided into risk categories, conventionally based on anatomical-surgical prognostic factors. The most significant prognostic factors are stage, histologic type, depth of myometrial invasion, grade of differentiation and lymph-node metastases [1]. Stage IC poorly differentiated, and stages II and III-IV show five-year survival rates ranging from approximately 20 to 60%, thus all requiring additional treatment [2]. Traditional prognostic factors, however, cannot define the prognosis for all patients. DNA content analysis can be useful to assess the risk of recurrence more precisely and ploidy appears to be one of the most important prognostic factors in EC [3-5].

There is much debate about the best adjuvant therapy after surgery and there are no accepted guidelines for this treatment. EC patients often receive adjuvant radiation therapy to reduce the risk of pelvic relapse [6,7] but this does not seem to improve overall survival because it cannot reduce the risk of distant recurrences. On the other hand, the efficacy of adjuvant chemotherapy alone has not been proved yet [8,9]. A new combined adjuvant treatment is therefore needed to improve results in high-risk EC patients.

Paclitaxel (P) has shown *in vitro *and clinical activity against EC and is also a potent radiosensitizer [10]. The goal of this trial was to try out a new regimen of combined chemotherapy and radiation therapy. The toxicity and safety of concomitant P and radiotherapy (RT) have already been evaluated [11] We report the results in 23 high-risk EC patients, meaning advanced stages, IC G3 and aneuploid tumors, treated with P and RT.

## Methods

Twenty-three patients with high-risk EC underwent primary surgery in our Institution or were referred just for adjuvant therapy. All were surgically treated with abdominal hysterectomy, bilateral salpingo-oophorectomy and peritoneal cytology; pelvic lymphadenectomy was done in 13 patients. In the other cases lymph node status was unknown because these patients were either referred to our Institution after primary surgery or randomized between pelvic lymphadenectomy and nodes sampling only if grossly involved. No systematic periaortic dissection was performed in these cases. Patients with macroscopic periaortic positive nodes were excluded.

Stages included IIB, IIIA (patients with positive washings without other unfavorable prognostic factors were omitted), IIIB and IIIC or IC G3 without lymphadenectomy or with aneuploid tumor (DNA index >1.2). Histologic types other than endometrioid adenocarcinoma were excluded. All the histological samples were examined by the same pathologist and in all the cases DNA ploidy was determined from paraffin-embedded tissue.

All the following criteria were satisfied: WHO performance status 0–1; adequate bone marrow reserve (neutrophil count >1.5, platelet count >100000 and Hb >10 g/dL), adequate liver function (serum bilirubin <1.5, serum transaminases < twice the upper limit of normal), adequate renal function (serum creatinine <1.5 mg/dL), no chronic cardiac or bowel diseases, age >18 years and <75 years. Patients with an history of other invasive cancer (except basal cell carcinoma of the skin) and previous chemotherapy or radiotherapy were excluded. The interval between surgery and RT had to be less than six weeks. Ethical approval was obtained from Ethical Committee of the Scientific Institution of San Raffaele Hospital of Milan.

Written informed consent was obtained from all patients before they entered the study.

P at a dose of 60 mg/m2 was infused intravenously in 250 mL of normal saline for 1 hour once weekly during RT for five weeks. Standard anaphylaxis premedication was given. The radiation plan consisted of a total dose of 50.4 [34] Gy, given in five fractions per week (1.8 Gy: daily dose) for six weeks. The irradiation field encompassed the entire pelvis as follows: the high limit was tangential to the upper surface of L5, the lower limit comprised the upper third of the vagina, and the lateral limits were not less than 2 cm outside the ileopectineal ligament.

Two IIIC patients underwent extended field irradiation. Brachytherapy was used in IIIB patient. Three further cycles of P, at a dose of 80 mg/m2, were given weekly at the end of RT.

Both chemotherapy and radiation therapy were delayed if, at the time of recovery, the neutrophil count was less than 1.0 × 10^9^/L or platelets were less than 100 × 10^9^/L. For all other toxicity we used the WHO toxicity scale. Chemotherapy was discontinued after two consecutive weeks of delay; when this happened patients continued RT alone.

Patients were assessed every three months for the first three years, every six months for the fourth and fifth year, then yearly.

Overall and disease-free survival were evaluated from the time of surgery. Survival rates was calculated according to intent to treat. Secondary we studied survival rates in the patients who completed at least five cycles of chemotherapy and all the RT in assessing the efficacy of this treatment.

Patterns of failure were recorded by the sites of failure: locoregional within the irradiated area, distant outside the irradiated area, or both.

## Results

The 23 patients with EC followed up for 16–48 months (median 34 months). Table 1 shows the main characteristics of the whole group. Five had metastatic lymph nodes. Six were stage IC, 4 stage IIB, 7 IIIA, 1 IIIB and 5 IIIC. Twenty-one patients had more than 50% myometrial infiltration; two stage IIIA cases only had less than 50% uterine infiltration. Two patients had well differentiated tumors, 8 were moderate tumors and 13 were poorly differentiated. DNA ploidy was determined in all patients on paraffin-embedded tissues: seven had aneuploidy (defined as a DNA index >1.2).

Two patients who stopped chemotherapy before the end of RT did not complete at least five cycles of chemotherapy. The first one had a tumor stage IIIA and presented an anaphylactic reaction to P at the first cycle and continued RT alone; she had a periaortic recurrence after six months and died of disease after 20 months of follow-up. The other, with a tumor stage IIB, refused to continue chemotherapy at the second cycle of P, without any toxicity, and died ten months later from a pericardial mesothelioma.

One-hundred and fifty-seven P cycles were administered both during radiotherapy and consolidation chemotherapy. Adverse effects were individually evaluated and recorded. There was no life-threatening toxicity. No patients required hospitalization or ER visits for acute toxicity. Adverse effects are summarized in Table 2.

No dose reduction was required. Eight cycles were delayed one week, in four cases for grade 3 neutropenia, in three for severe diarrhea and one for increased liver enzymes. No patients presented emesis.

Hematological toxicity was mild in other cases without reduction of doses or delay of treatment. No blood transfusions or hematologic support were administered in these patients. One patient developed anaphylactic clinical reaction and suspended the chemotherapy. No patients developed alopecia.

Delayed toxicity included one case of incomplete small intestinal obstruction 8 weeks after the end of the treatment with medical resolution. Three patients reported mild recurrent intestinal dysfunctions (stipsis and/or diarrhea). One patient presented recurrent cystitis related to the treatment.

Relapses occurred in five patients (21.7%). Median time to recurrence was 18.6 months (range 3–28). None of the five stage IIIC patients with metastatic pelvic nodes developed periaortic recurrences (Table 3).

Final event, defined according to date of death or last contact, showed 17 patients (73.9%) still alive and free from disease, one alive with tumor, four dead for disease. Another patient died from another primary tumor.

Overall survival of the patients who completed chemo-radiation was 81%. In this group median time to recurrence was 19.2 months. All recurrences were outside the radiation field. One patient presented a periaortic and pulmonary relapse 28 months after surgery and died 45 months from diagnosis; one had an abdominal relapse after 25 months and died 48 months from surgery. The third developed a periaortic nodule, outside the radiation field, after three months and died after 18 months of follow-up. The fourth patient had a skeletal relapse after 21 months but is alive after 26 months of follow-up. All these patients completed 8 cycles of chemotherapy in combination with radiotherapy. The patients with cancer relapse were treated in four cases with chemotherapy (Cisplatin and Doxorubicine) and in one case with hormonal treatment.

## Discussion and conclusion

Although there have been improvements in RT and chemotherapy, during the last few years survival for high-risk EC has not increased. Reported five-year survival rates are 70% for stage II, 50% for stage III, and 27% for stage IV [12]. This has prompted the search for new therapeutic strategies in recent decades. Looking at the literature it seems that surgery alone could be the treatment of choice for low-risk EC but more aggressive treatments are appropriate for high-risk EC.

The role of adjuvant RT in the treatment of EC has remained controversial due to a lack of information from randomized trials. Retrospective analysis suggested that the risk of locoregional relapse was reduced but overall survival rates did not seem to be improved. Three randomized trials comparing no therapy vs RT showed a reduction of pelvic relapses in the RT patients but not in overall survival rates. did not change [13-15].

Whole abdominal radiation therapy has also been employed in particular in the treatment of advanced endometrial cancer; Sutton et al found 29% recurrence free survival in patients with endometrial adenocarcinomas stage III-IV with gross residual disease, gross extrauterine spread with all disease resected or microscopic extrauterine disease [16] while Martinez et al obtained 59% disease free survival rate in stage I-III endometrial carcinoma at high risk for intra-abdominopelvic recurrence [17]. The comparison between whole abdominal radiation therapy and Doxorubicin plus Cisplatin chemotherapy in a randomized phase III trial on advanced endometrial carcinoma showed a significantly improved progression-free and overall survival in patients treated with chemotherapy [18].

Systemic chemotherapy has been investigated in an effort to improve the outcome of advanced or recurring EC [19,20]. Doxorubicin and Cisplatin have been the most frequently used cytotoxic drugs. A few non-randomized trials using Doxorubicin/Platinum-based regimens have suggested that chemotherapy may be beneficial in some patient subsets, and response rates of 42% and 38% are reported [21,22].

Though larger-scale randomized trials are still few, post-operative chemotherapy alone seemed to be more effective in the control of distant recurrence than in pelvic relapses. Mundt et al. [23], in a retrospective study, evaluated the efficacy of adjuvant chemotherapy on 43 high-risk EC patients and reported a pelvic relapse rate of about 50%.

In a series of patients submitted to adjuvant chemotherapy without locoregional RT, Tsunoda et al. [24] noted recurrences in 25%, all within the pelvis. Fujimura et al. [25] reported 15 recurrences in 25 high-risk patients treated with adjuvant chemotherapy alone; 53% were in the pelvis.

In the CNR study [8] EC patients were divided into low, medium and high-risk groups. The 339 high-risk evaluable patients (IC G3; IIA and IIB G3; stage III) were randomized to pelvic post-operative RT or adjuvant chemotherapy with PAC (Cisplatin, Adriamycin and Cyclophosphamide) for five cycles. Overall survival was not significantly different in the two groups. However, GOG 122, a randomized trial comparing Doxorubicin/Platinum versus radiation, found improvement in survival and progression-free survival (PFS) in patients with stage III-IV EC treated with chemotherapy [26].

For better control of both pelvic and distant failures, combinations of chemotherapy and RT have been tested. The GOG trial evaluated adjuvant Doxorubicin after surgery and RT for high-risk EC in a randomized prospective manner. After completion of RT, patients were randomized to either the Doxorubicin treatment arm or no further treatment; there was no significant difference in survival and progression-free interval between the two arms [27].

Platinum-based chemotherapy followed by RT has also been used. O'Brien, in a series of 26 high-risk EC patients treated with chemotherapy (PAC) and RT, reported 57.8 % recurrences [28]; this result was similar to a non-randomized group of patients treated with sequential chemotherapy and RT, in which there were 49% recurrences [29]. Schorge reported five-year survival of about 50% in stage III EC patients assigned different treatments [30]. Gabriele [31], in a series of high-risk EC patients (19 stage III, 2 IV) treated with 3–5 cycles of PAC followed by RT, reported an overall incidence of recurrence of 57.1%, similar to other studies. These data show that Platinum and Doxorubicin chemotherapy followed by RT is feasible and well tolerated, but the impact of chemotherapy before adjuvant external RT on survival has not been demonstrated in high-risk EC.

P was introduced for the treatment of EC after its success in ovarian and breast cancers. Three trials achieved response rates of 36–43% when P was used as single agent [32-34]. P has also shown activity in platinum-resistant patients [32]. Hoskins [35] tested P and carboplatin, alone or followed by RT, in a phase II study in advanced and recurrent EC patients, obtaining response rates of 78% and 56% respectively in the two groups; toxicity was manageable, reversible and mainly hematological.

We analyzed the combination of P and pelvic RT as adjuvant treatment in high-risk EC patients. We chose P because recent studies have indicated its efficacy as single agent in EC [32-34]. The drug acts by enhancing microtubular assembly and preventing microtubular depolymerization [36]. Another mechanism of action is of particular interest for radiochemotherapy. P is the first member of the taxane family, which recruits cells in the G2/M phase of the cell cycle, in which cells are more sensitive to the killing effects of ionizing radiation [37]. Other radiosensitizing actions include inhibition of the tumoral cells' repair capacity, lower hypoxic resistance, depletion of sulfhydril groups, and effects on the cell membrane [38]. The weekly P regimen was planned to optimize its radiosensitizing action.

Several studies have defined the toxicity of concomitant P and RT. The possibility of using P, at dosages ranging from 40 to 80 mg/m2, together with RT has already been demonstrated in other tumors, without serious adverse effects [39,40]. In our study we employed a 60 mg/m2 dosage of P without severe side effects, except for one allergic reaction.

Weekly P in association with pelvic RT is a well tolerated regimen. In addition, we did not observe any recurrence within the pelvis, whereas two other Italian studies [31,41] reported high rates of pelvic relapse after sequential chemoradiation therapy. These data support the radiosensitizing effect of P and suggest that a delay in starting RT might reduce its therapeutic effect. Greven [42], in a preliminary analysis of adjuvant combined chemoradiotherapy, also reported a low relapse rate. Concomitant chemoradiation seems to achieve adequate pelvic control. Comparison in recurrence rates between different kind of postoperative treatments in endometriale cancer have been reported in table 4.

Considering that in our series all the recurrences were outside the radiation field it might be useful to use a higher dose of Taxol in consolidation cycles or to add another active agent with a different toxic profile to improve survival in high-risk EC, and ensure better systemic control.

In conclusion, this series is obviously too small to prove that concomitant RT and P do actually reduce relapse rates and improve overall survival. Nevertheless, these encouraging results confirm the enhancing effect of this approach in high-risk EC. Longer follow-up is now needed to assess the outcome and randomized trials are required.

## Abbreviations

endometrial carcinoma (EC), radiotherapy (RT), paclitaxel (P)

## Competing interests

The author(s) declare that they have no competing interests.

## Authors' contributions

MG conceived of the study, and participated in its design and coordination.

DP and VR participated in drafting the manuscript.

BS carried out all the radiation treatment and the patients' follow-up.

RE and GC participated in the acquisition and analysis of data.

FL and FF participated in revising critically the manuscript.

## Pre-publication history

The pre-publication history for this paper can be accessed here:



## Figures and Tables

**Table 1:** Patients' main characteristics

	**Age**	**Grade**	**Myometrial invasion**	**Ploidy**	**Stage**	**Lymph node status**
1	54	3	2	1.00	IIB	Negative
2	50	2	2	1.00	IIIA	Negative
3	67	3	2	1.00	IIB	Not done
4	67	3	2	1.00	IC	Not done
5	64	2	2	1.00	IIIC	Positive
6	67	3	2	1.30	IIIA	Not done
7	63	2	2	1.50	IIIC	Positive
8	48	3	2	1.30	IC	Negative
9	62	2	1	1.00	IIIA	Not done
10	55	2	2	1.00	IIIC	Positive
11	62	3	2	1.00	IC	Not done
12	69	3	2	1.60	IC	Not done
13	71	2	2	1.00	IIIA	Negative
14	64	1	2	1. 00	IIIA	Negative
15	71	2	2	1.90	IIIC	Positive
16	63	3	2	1.80	IIIC	Positive
17	71	3	2	1.00	IC	Not done
18	74	3	2	1.50	IIIB	Negative
19	65	2	2	1.00	IIIA	Not done
20	61	3	2	1.00	IIB	Negative
21	64	3	2	1.20	IIB	Negative
22	51	1	1	1.00	IIIA	Not done
23	61	3	2	1.00	IC	Not done

**Table 2:** Toxicity (WHO scale)

	**Grade 1**	**Grade 2**	**Grade 3**	**Grade 4**
**Leukopenia**	1	15	2	-
**Neutropenia**	7	3	4	-
**Thrombocytopenia**	-	-	-	-
**Anemia**	7	2	-	-
**Gastrointestinal**	1	2	1	-
**Liver**	-	1	-	-
**Cardiac**	-	-	-	-
**Neuropathy**	-	-	-	-
**Cystitis**	1	2	-	-
**Alopecia**	-	-	-	-
**Emesis**	-	-	-	-

**Table 3:** Site of failure

**Patient**	**Site of relapse**	**Stage**	**Survival (months)**	**Node**
**1**	Lung-aortic node	IIIA	45 DOD	Not done
**2**	Abdomen	IC	48 DOD	Negative
**3**	Aortic nodes	IC	18 DOD	Negative
**4**	Bone	III C	26 AWT	Positive
**5**	Aortic nodes	IIIA	20 DOD	Not done

DOD: dead of disease

AWT: alive with tumor

**Table 4:** Post-operative treatments and recurrence rates in endometriale cancer: comparison between current study and literature data.

	**Stage**	**N patients**	**Treatment**	**Recurrence %**	**Pelvic or extrapelvic + pelvic recurrence**
**Mundt (22)**	I-IV	43	Chemotherapy	67.4 %	49.3%
**Smith (28)**	IC-IV	39	CAP/RT	38.5%	20%
**Gabriele(30)**	III-IV	21	CAP/RT	57%	50%
**Bruzzone**	III-IV	45	CAP/RT	40%	77.8%
**Martinez**	I-III	132	HDWART	35%	59%
**Greven**	IC-III	46	Chemo/RT*	22%	30%
**Current study**	ICG3-III	23	Chemo/RT+	21.7%	None

CAP/RT : Cisplatin, doxorubicin and cyclophosphamide followed by radiotherapy HDWART high-dose abdomino-pelvic irradiation ^ abdominal/pelvis and vagina recurrence

Chemo/RT concomitant chemo-radiation *cisplatin days 1/28 followed after radiotherapy by cispaltin and paclitaxel

+concomitant radiotherapy with paclitaxel
